# Preparation and Biochemical and Microbial Behavior of Poly(Lactide) Composites with Polyethersulfone and Copper-Complexed Cellulose Phosphate

**DOI:** 10.3390/ma18132954

**Published:** 2025-06-22

**Authors:** Marcin H. Kudzin, Zdzisława Mrozińska, Anna Kaczmarek, Jerzy J. Chruściel, Martyna Gloc, Renata Żyłła

**Affiliations:** 1 Łukasiewicz Research Network—Lodz Institute of Technology, 19/27 Marii Sklodowskiej-Curie Street, 90-570 Lodz, Poland; zdzislawa.mrozinska@lit.lukasiewicz.gov.pl (Z.M.); anna.kaczmarek@lit.lukasiewicz.gov.pl (A.K.); jerzy.chrusciel@lit.lukasiewicz.gov.pl (J.J.C.); martyna.gloc@lit.lukasiewicz.gov.pl (M.G.); renata.zylla@lit.lukasiewicz.gov.pl (R.Ż.); 2 Department of Bioprocess Engineering, Faculty of Process and Environmental Engineering, Lodz Univeristy of Technology, 213 Wolczanska Street, 90-924 Lodz, Poland

**Keywords:** activated partial thromboplastin time (aPTT), prothrombin time (PT), antimicrobial properties, biological activity, blood coagulation, cellulose phosphate, composite, copper, melt-blown, polyethersulfone, poly(lactide) (PLA)

## Abstract

This research investigates the biochemical and microbiological characteristics of a composite comprising poly(lactide) (PLA) combined with polyethersulfone (PESf) and copper-complexed cellulose phosphate (CelP-Cu). The material was produced using the pneumothermic melt-blown method and then modified with polyethersulfone and cellulose phosphate, followed by complexation with copper ions using the dip-coating technique. Comprehensive physicochemical and biological evaluations were conducted to characterize the composite. The physicochemical assessments involved elemental analysis (C, O, Cu) and morphology examination. The biological evaluations encompassed microbiological testing and biochemical–hematological analysis, including activated partial thromboplastin time (aPTT) and prothrombin time (PT). Antimicrobial activity was assessed according to the EN ISO 20645:2006 and EN 14119:2005 standards, by placing material specimens on agar plates inoculated with representative microorganisms. The results revealed that the composites exhibited significant antimicrobial effects against model microorganisms: *Staphylococcus aureus*, *Escherichia coli*, *Klebsiella pneumoniae*, *Pseudomonas aeruginosa*, *Bacillus atrophaeus*, *Candida albicans*, *Saccharomyces cerevisiae*, *Aspergillus niger*, *Chaetomium globosum*. This study highlights the potential of PLA/PESf/CelP-Cu composites for novel biomedical applications, demonstrating their biocompatibility and their influence on hemostatic processes and antimicrobial properties.

## 1. Introduction

Polymers constitute a very promising group of materials used in a variety of different fields, including biomedical and healthcare applications like tissue engineering, wound management, and biotechnology [1,2,3,4]. As the demand for innovative solutions continues to grow, different hybrid polymer materials are becoming more and more popular [1,2,3,4]. In particular, composite materials with enhanced functional properties are currently being vastly researched.

Among the various synthetic polymers applied in biomedicine, poly(lactide) (PLA) is gaining a lot of attention [5,6,7,8,9]. It has a wide range of applications in biomedicine and healthcare, particularly in drug delivery systems, wound management, as well as in regenerative medicine [9,10,11,12,13,14,15,16,17,18]. PLA is a biodegradable polymer, which degrades primarily through hydrolysis, and the byproducts of this process can be removed through natural biological mechanisms, ensuring no negative impact [5,8,9]. Moreover, it is characterized by good physical and mechanical properties [8,19], biocompatibility [20], nontoxicity [19,20], thermoplastic processability, and eco-friendliness [9,21,22]. Nonetheless, it possesses certain disadvantages, including inadequate toughness, hydrophobic characteristics, and the absence of reactive side chain groups, which restrict its applicability [21]. Thus, combining PLA with other polymers, such as, for example, polyethersulfone (PESf), offers a simple and efficient approach to enhance specific properties of the material [21].

Polyethersulfone is a linear, amorphous, and thermoplastic polymer [1,23] with excellent film-forming properties [21,24]. Due to its good biochemical properties, it finds application in various medical devices, especially for blood purification, i.e., hemodialysis, hemofiltration, plasmapheresis, and plasma collection [1,21,23,25,26]. It is characterized by relatively low flammability [23] and poor water sorption [1,23]. PESf possesses exceptional mechanical properties [21,24,25], favorable stiffness due to the presence of sulfone group, and desirable flexibility owing to the ether bonds [1]. Moreover, it exhibits an outstanding thermal stability, as well as chemical stability [1,21,25], which may be attributed to the presence of aromatic rings [1] and a high glass transition temperature [23]. According to the literature, polyethersulfone shows good resistance towards various organic solvents, acids, bases, as well as UV radiation [1]. Nevertheless, its use may be limited as a result of the intrinsic hydrophobicity, causing biofouling and insufficient hemocompatibility [1,25]. Upon contact with blood, proteins are adsorbed to the surface of PESf and cause platelet aggregation and activation, which in turns induces blood coagulation [1,23]. Therefore, different modification methods have been developed in order to enhance the compatibility of blood with PESf [23,24,25,26]. For example, in order to minimize the blood immune response, different biomimetic, zwitterionic, non-ionic, anticoagulant molecules, and hydrophilic brushes were immobilized or blended with PESf. These additives modified the nature of the membrane, enhanced its biocompatibility, and also improved the uremic waste dialysis properties [23]. Alternatively, one surface of the PESf membrane was modified with a novel triblock amphiphilic copolymer of poly(styrene-co-acrylic acid)-*b*-poly(vinyl pyrrolidone)-*b*-poly(styrene-*co*-acrylic acid). The surface segregation led to a PESf matrix with hydrophilic and anionic properties, resulting in improved blood compatibility [24]. However, it is difficult to control the surface structure of the PESf membranes, and characterization methods do not yet allow the observation of the dynamic adsorption behavior of various proteins on the membranes’ surfaces. The hydrophilicity, lowered protein adsorption, blood compatibility, cytocompatibility, and suppressed platelet adhesion of the membrane prepared by blending sulfonated polyethersulfone with poly(acrylonitrile-*co*-acrylic acid-*co*-vinyl pyrrolidone) were improved by modifying the surface of the membrane with a heparin [26]. The further development of the next generation of PESf-based hemodialyzers, bio-artificial liver supports, external artificial kidneys, and blood purification instruments still remains a great challenge [25,26].

One of the possible additives to composite polymeric biomaterials may be cellulose phosphate. Phosphorylated cellulose demonstrates advantageous physicochemical properties, such as biocompatibility, nontoxicity, hydrophilicity, biodegradability, and high ion exchange capacity [27,28,29,30]. Additionally, it is characterized by the crosslinking ability with other polysaccharides and binding ability with biologically active species through negative charges present on phosphate groups [27]. Thanks to the abovementioned properties, cellulose phosphate is applicable in the biomedical field, for example, in drug delivery systems. One of the unique advantages of cellulose phosphate is its extraordinary binding ability to calcium ions and growth factors [27,29]. As a result, cellulose phosphate is believed to stabilize blood and may serve as a hemostatic agent [31]. The anticoagulant potential of cellulose phosphate was confirmed by Groth et al. [32]. Moreover, phosphorylated cellulose is capable of enhancing the bioactivity of polymers due to the presence of phosphate groups, which are able to induce apatite layer formation and mineralization [33]. Thus, cellulose phosphate is also a promising material in terms of bone tissue engineering [34]. Finally, our previous work has also proven that cellulose phosphate may serve as an antimicrobial agent when complexed with copper [35].

Copper is receiving increasing recognition among various antimicrobial agents [36,37,38,39,40,41,42,43]. The literature has thoroughly documented the biocidal properties associated with copper and its complexes [38,39,40,41,42,43,44,45,46]. The antibacterial efficacy of copper’s metallic surface may be attributed to two primary mechanisms: the direct contact between copper and bacteria (referred to as “contact killing”) and the oxidation of copper, which results in the release of copper ions [41,47,48,49,50,51,52,53].

As reported in the literature, the phenomenon known as “contact killing” is a result of cellular damage due to the direct physical interaction between microorganisms and surfaces coated with metallic copper [54]. This process is connected with the accumulation of copper ions released from the surface in the membrane of the microbes [54,55]. The resulting membrane destabilization, caused by a drop in its potential, ultimately results in the damage of the membrane [54,55]. Consequently, copper ions penetrate and accumulate within the cell [54,55], initiating a series of subsequent effects. Initially, reactive oxygen species, particularly hydroxyl radicals, are produced through Fenton-like reactions [54,55,56]. These newly generated free radicals may cause the oxidation of lipids and proteins [56]. Additionally, they have the potential to damage the structure of DNA, resulting in its degradation [54,55]. The copper ions released during this process can react with the metal-binding sites of various enzymes, leading to their inhibition [54,55,56]. For instance, Cu ions can adversely affect Fe–S clusters in cytoplasmic hydratases by binding to sulfur and subsequently displacing iron [54,55,56]. This process leads to a decrease in sulfhydryl groups, including cysteine in glutathione [54,56]. In addition, competition of copper ions with other metal ions for divalent cation-binding sites on proteins may occur [56]. As well as that, the presence of Cu ions can adversely affect microbial respiration, potentially leading to its suppression due to the inhibition of cytochromes [54,56].

Simultaneously, copper demonstrates comparatively lower toxicity than other heavy metals, including silver [40,57,58]. Despite the high sensitivity of microorganisms to copper, human tissues do not display the same susceptibility [43,57,58]. Additionally, copper occurs naturally in human tissues and is an essential element that serves multiple critical functions [39,41,57,59]. Consequently, the deficit of copper is regarded as more concerning in comparison with its toxicity [39], owing to the ability of the human body to efficiently metabolize copper. Moreover, there are various mechanisms that provide protection against copper toxicity at the cellular, tissue, and organ levels [39]. Furthermore, copper is much more cost-effective compared with silver, which is one of the most prevalent antimicrobial agents [41,59].

Furthermore, copper is also regarded as an important factor in wound management, significantly contributing to wound healing [60,61,62]. Research indicates that copper promotes the vascular endothelial growth factor (VEGF) production and thus facilitates angiogenesis [60,61,62,63]. Additionally, it has been reported that copper elevates the expression of integrin, which supports the stabilization of fibrinogen and collagen [60,61,62]. Furthermore, copper is crucial for matrix remodeling, cell proliferation, and re-epithelization as it enhances the activity of copper-dependent enzymes, proteins, and polysaccharides [61,62,64]. The beneficial effects of copper on the process of wound healing have been well documented in the literature [60,62,65,66]. Finally, some studies also suggest that Cu^2+^ ions may influence the intrinsic blood coagulation pathway as they adsorb contact factors (XI, XII, HK), which leads to a decrease in their concentration in plasma [67].

In this study, we developed a novel composite material composed of poly(lactide) (PLA), polyethersulfone (PESf), and copper-complexed cellulose phosphate (CelP-Cu). The objective was to assess the potential of this composite for various biomedical applications. Specifically, the research aimed to evaluate the composite’s ability to inhibit microbial growth; examine its impact on blood coagulation factors; and ultimately determine its suitability for wound management, tissue engineering, and other biomedical fields that require antimicrobial properties and improved biocompatibility.

## 2. Materials and Methods

### 2.1. Materials

- Poly(lactic acid) (PLA): The polymer was obtained from NatureWorks LLC (Minnetonka, MN, USA), type Ingeo™ Biopolymer 3251D, MFR = 30–40 g/10 min (190 °C/2.16 kg), T_m_ = 160–170 °C, glass transition temperature 55–60 °C, weight average molecular weight M_w_ = 55,400 g/mol, polydispersity index (PDI): M_w_/M_n_ = 1.62, in the form of a granulate and was used for the fabrication of nonwoven samples.
- Reagents used for the preparation of composites: polyethersulfone (PESf) Ultrason E6020P from BASF (Ludwigshafen am Rhein, Germany)—weight average molecular mass M_w_ = 72,000 g/mol (by GPC in DMAc, PMMA standard), PDI (M_w_/M_n_ = 3.5) (producer’s data); polyvinylpyrrolidone (PVP) from Sigma-Aldrich (St. Louis, MO, USA)—average M_w_ ~29,000 g/mol (producer’s data); 1-methyl-2-pyrrolidone (NMP) from Chemland (Stargard, Poland); cellulose phosphate from Sigma-Aldrich (St. Louis, MO, USA); and copper(II)chloride from Sigma-Aldrich (St. Louis, MO, USA).
- Microorganisms used in the analyses: *Staphylococcus aureus* (ATCC 6538), *Escherichia coli* (ATCC 11229), *Klebsiella pneumoniae* (ATCC 4352), *Pseudomonas aeruginosa* (ATCC 27853), *Bacillus atrophaeus* (ATCC 9372), *Candida albicans* (ATCC 10231), *Saccharomyces cerevisiae* (ATCC 9763), *Aspergillus niger* (ATCC 6275), *Chaetomium globosum* (ATCC 6205) were purchased from Microbiologics (St. Cloud, MN, USA).
- Standard human blood plasma lyophilizates (Dia-CONT I), aPTT reagent (Dia-PTT), PT reagent (Dia-PT), and 0.025 M CaCl_2_ solution reagent were obtained from Diagon Kft (Budapest, Hungary), as well as a coagulometer (K-3002 OPTIC, KSELMED^®^, Grudziądz, Poland), were utilized for the experiments. All the reagents were prepared in accordance with the manufacturer’s instructions.

### 2.2. Methods

#### 2.2.1. Preparation of PLA/PESf/CelP-Cu Composites

##### Poly(Lactic Acid) Nonwoven Fabrics

Nonwovens made of poly(lactic acid) were produced using the melt-blown method. A laboratory extruder with a single screw (Axon, Limmared, Sweden) was employed, featuring a head with 30 holes, each having a diameter of 0.25 mm. The processing parameters for the fabrication of the poly(lactic acid) nonwoven samples are listed in Table 1.

##### Poly(Lactic Acid) Nonwoven Fabric Coating Procedure

**Figure 1:**
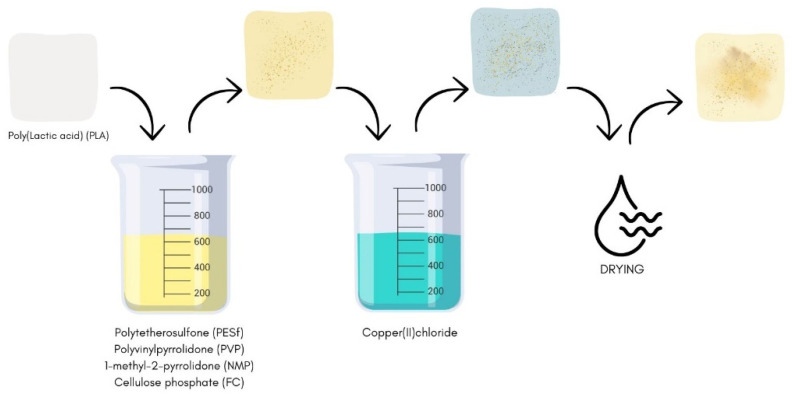
The schematic diagram of the procedures for surface modification of poly(lactic acid) material.

The poly(lactic acid) nonwoven samples were modified through a dip-coating, two-step method (Figure 1).

##### Polymer Solution

Polyethersulfone (PESf) in the form of white flakes, polyvinylpyrrolidone (PVP), and the magnetic dipole were placed in an Erlenmeyer flask under a reflux condenser. The flask was then placed on a magnetic stirrer with heating function (Chemland, Stargard, Poland). The temperature was set to 100 °C, with the initial stirring temperature 22 °C. After starting the stirrer, the solvent (NMP) was added. The applied high temperature was necessary due to the high viscosity of the dissolving polymer, and the stirring speed was adjusted according to the viscosity. After the dissolution process, the solution containing 14 wt.% of PESf and 2 wt.% of PVP was allowed to cool down and equilibrate to ambient temperature, followed by the addition of cellulose phosphate (CelP). The mixture was then placed back on the magnetic stirrer for 1 h. The final polymer exhibited a slightly yellow, transparent color and a uniform consistency.

##### Composite Preparation

**Figure 2:**
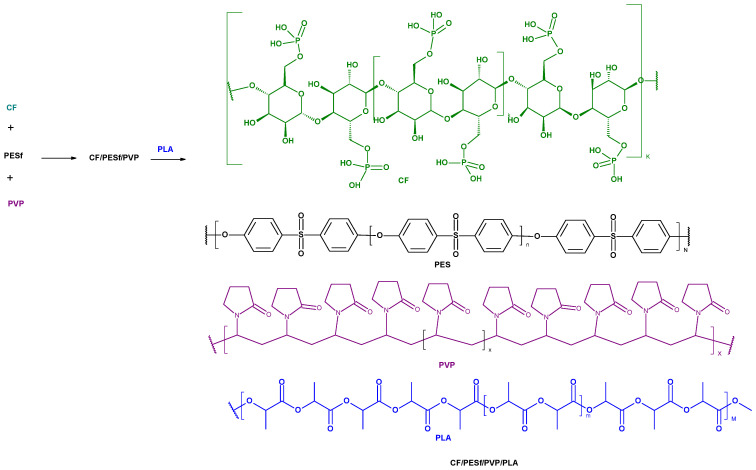
The putative mechanism of PLA/PESf/CF composite formation.

**Figure 3:**
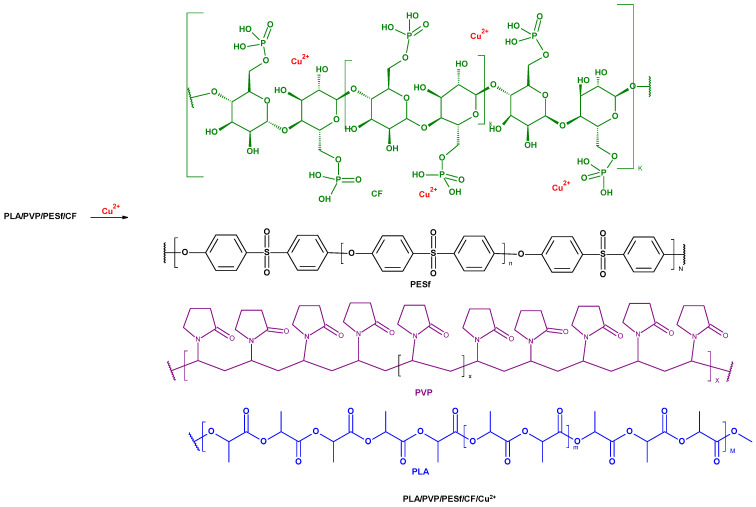
A visualization of the putative mechanism of copper deposition on the surface of PLA/PVP/PESf/CelP composites (PLA/PVP/PESf/CelP → PLA/PVP/PESf/CelP/Cu^2+^).

Samples of poly(Lactic acid) nonwoven (PLA) were impregnated (at room temperature, within 1 min each step): (1) in a homogeneously dispersed dispersion of CelP (5 wt.%) in the polymer solution (PESf, 14 wt.% + PVP, 2 wt. %), and (2) then this way the impregnated PLA samples were immediately transferred into two different aqueous solutions of copper (II) chloride (with concentrations: 1 wt.% and or 10 wt.%), and next reimmersed. We simply used a tweezers in order to transfer impreganted nonwoven PLA samples, previously impregnated with PESf/PVP solutions, into the CuCl2 solutions with different concentrations. It seems that PVP plays a role of compatibilizer or/and plasticizer in the structure of the complex, multilayer polymeric composite (Figure 2 and Figure 3).

The PLA/PESf/CelP-Cu samples were then squeezed and next were dried at 40 °C for 6 h, i.e., until a constant weight was achieved. As a reference, a poly(lactic acid) nonwoven sample modified using the same polymer concentrations, but without the copper solution modification, was prepared (sample PLA/PESf/CelP). Table 2 presents the abbreviations of the poly(lactic acid) nonwoven samples used in this investigation.

#### 2.2.2. Copper Content in Composite Materials by Flame Atomic Absorption Spectrometry (FAAS)

The copper content in the PLA/PES/CelP-Cu composites was determined after sample mineralization using a Magnum II microwave mineralizer (Ertec, Wroclaw, Poland). Flame atomic absorption spectrometry was then employed to measure the copper concentration in the composite samples. The copper concentration was measured with a Thermo Scientific Solar M6 atomic absorption spectrometer (LabWrench, Midland, ON, Canada). The spectrometer was equipped with single-element coded hollow cathode lamps and a 100 mm titanium burner. The background correction mechanism was applied using a D2 deuterium lamp. The total copper content M [mg/kg; ppm] in the PLA/PES/CelP-Cu composite samples was calculated using Equation (1) [68]:

(1) $$M=\frac{C\cdot V}{m}$$

where

*C*—metal concentration in the mineralized PLA/PESf/CelP-Cu sample solution [mg/L];
*m*—mass of the mineralized sample of PLA/PESf/CelP-Cu composites [g];
*V*—volume of the sample solution [mL].

#### 2.2.3. Analysis of Surface Morphology

To assess the morphology and structure of the samples at lower magnifications, optical microscopy was used. This imaging was carried out with a VHX-7000N digital microscope from Keyence (Osaka, Japan). Scanning electron microscopy (SEM) was employed to examine the surface morphology and structure of the obtained composites in more detail. The morphological features were observed using a scanning electron microscope—Phenom ProX G6 (Thermo Fisher Scientific, Waltham, MA, USA)—operating in a low-vacuum environment (60 Pa) with an accelerating voltage equal to 15 keV. The back-scattered electron detector was utilized. For qualitative elemental analysis, the energy-dispersive X-ray spectroscopy (EDS) unit from Oxford Instruments (Abingdon, UK) was used.

#### 2.2.4. Evaluation of Antimicrobial Activity

The antibacterial and antifungal properties of the composites were evaluated following the standards EN ISO 20645:2006 (for antibacterial properties) [69] and EN 14119:2005 (for antifungal properties) [70]. Specimens (10 mm × 10 mm) of the tested material (unmodified PLA, PLA/PES/CelP, and PLA/PES/CelP-Cu) were placed on inoculated agar plates (pH: 6.2). The samples were incubated at 37 °C for 24 h in the case of bacteria strains and at 30 °C for 14 days in the case of fungi. The agar was inoculated with model bacterial and fungal strains: *Staphylococcus aureus* (1.9 × 10^8^ CFU/mL), *Escherichia coli* (2.6 × 10^8^ CFU/mL), *Klebsiella pneumoniae* (1.1 × 10^8^ CFU/mL), *Pseudomonas aeruginosa* (1.5 × 10^8^ CFU/mL), *Bacillus atrophaeus* (0.7 × 10^8^ CFU/mL), *Candida albicans* (0.8 × 10^7^ CFU/mL), *Saccharomyces cerevisiae* (2.3 × 10^7^ CFU/mL), *Aspergillus niger* (1.6 × 10^6^ CFU/mL), *Chaetomium globosum* (2.2 × 10^6^ CFU/mL). The antimicrobial activity of the composites was assessed by examining microorganism growth in the contact zone between the agar and the specimen, on the surface of the specimens, and by determining inhibition zones around the specimens. All the tests were performed in duplicate.

#### 2.2.5. Evaluation of Activated Partial Thromboplastin Time (aPTT) and Prothrombin Time (PT) Measurements

Frozen and lyophilized human plasma was dissolved in deionized water. For testing, 1 mg of the sample was immersed in 200 µL of plasma, followed by centrifugation and incubation for 15 min at 37 °C. The aPTT was determined by means of the Dia-PTT reagent, containing kaolin and cephalin, in combination with a 0.025 M CaCl_2_ solution. Firstly, 50 µL of Dia-PTT reagent was added to 50 µL of plasma and then incubated at 37 °C for 3 min. Next, 50 µL of 0.025 M CaCl_2_ was added to trigger the coagulation. To measure the PT, 50 µL of plasma was incubated for 2 min at 37 °C. Afterwards, 100 µL of Dia-PT reagent was added. The Dia-PT reagent, containing rabbit brain thromboplastin, calcium ions, and a preservative, was thoroughly mixed before use. The aPTT and PT were measured using the K-3002 OPTIC coagulometer (KSELMED^®^, Grudziądz, Poland).

## 3. Results and Discussion

### 3.1. Physical Properties of PLA/PES/CelP-Cu Composites

#### 3.1.1. FAAS Analysis for Copper Content and Surface Properties

The concentration of copper in the PLA/PES/CelP-Cu samples was evaluated by means of flame atomic absorption spectrometry (FAAS) after sample digestion. The results are presented in Table 3.

A noteworthy observation is the proportional, almost linear increase in the copper content in the composite samples (0.9155 and 9.5585 mg/kg) with the increasing concentration of the CuCl_2_ used in the dip-coating bath (1 and 10%).

#### 3.1.2. Surface Characteristics

**Figure 4:**
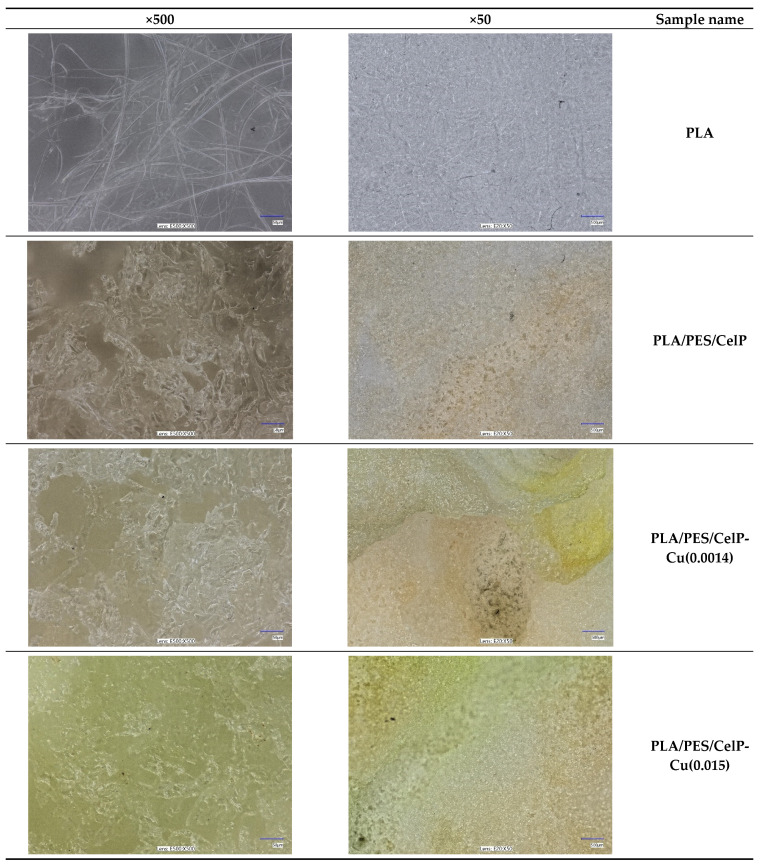
Optical microscopy images (magnifications: ×50; ×500) showing the surface morphology of the PLA/PES/CelP-Cu composites.

**Figure 5:**
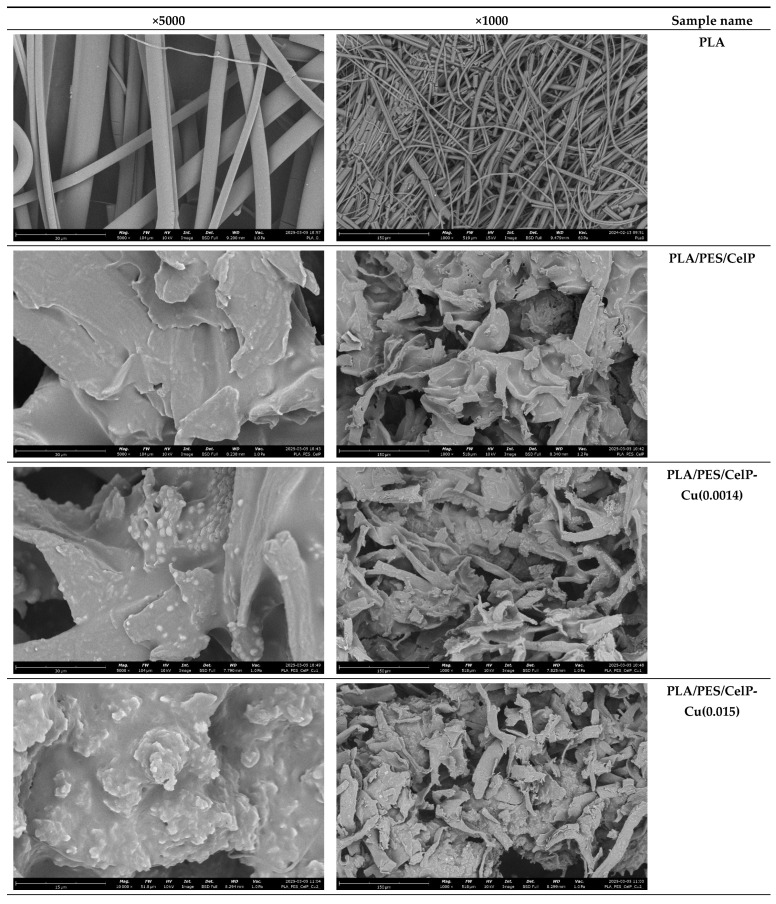
SEM results (magnifications: ×1000, ×5000) of the samples prior to (PLA) and after the coating process (PLA/PES/CelP-Cu).

Figure 3 and Figure 4 show the morphology of the samples’ surface, both prior to and after the modification process, examined using the optical microscopy and scanning electron microscopy (SEM) at various magnifications. The images clearly highlight the successful application of the coating, evident in the notable color change (Figure 4). The coating layer is visible not only on the outer fibers of the fabric but also on the inner fibers, penetrating deeper into the material (Figure 5).

In the case of the unmodified PLA nonwoven samples, the characteristic 3D (three-dimensional) fibrous structure with interconnected pores may be observed. The observed fibers are randomly oriented and differ significantly in size (both in length and in diameter), what is typical for melt-blown nonwovens. The surface of the unmodified PLA fibers is rather smooth (Figure 5).

The modification of PLA nonwovens with the subsequent coating using the polymer solution and CuCl_2_ solution resulted in a significant change in the morphology and structure of the samples due to the presence of either the PES/CelP or the PES/CelP-Cu coating on the surface (Figure 4 and Figure 5). As a result of the presence of the coating around the PLA fibers and in between the fibers, fewer pores are visible (Figure 4 and Figure 5). Moreover, the individual fibers are less distinguishable due to the overlap effect (Figure 3 and Figure 4), while the fibers’ surface is less smooth (Figure 5).

For the PLA/PES/CelP-Cu samples, numerous agglomerates are visible on the entire surface (Figure 4). The lack of the observed agglomerates in the case of the PLA/PES/CelP composite indicates that these are the CuCl_2_ agglomerates. This is consistent with the fact that the amount of the agglomerates significantly increases (Figure 4) with the rise in the content of the CuCl_2_ in the modifying mixture, i.e., for the PLA/PES/CelP-Cu(0.015) sample. In consequence, the surface of the modified PLA/PES/CelP-Cu samples is more rough and less uniform.

As a result of the coating procedure, the fibrous structure of the samples was partially deformed; however, the overall 3D structure of the PLA nonwoven was preserved.

**Figure 6:**
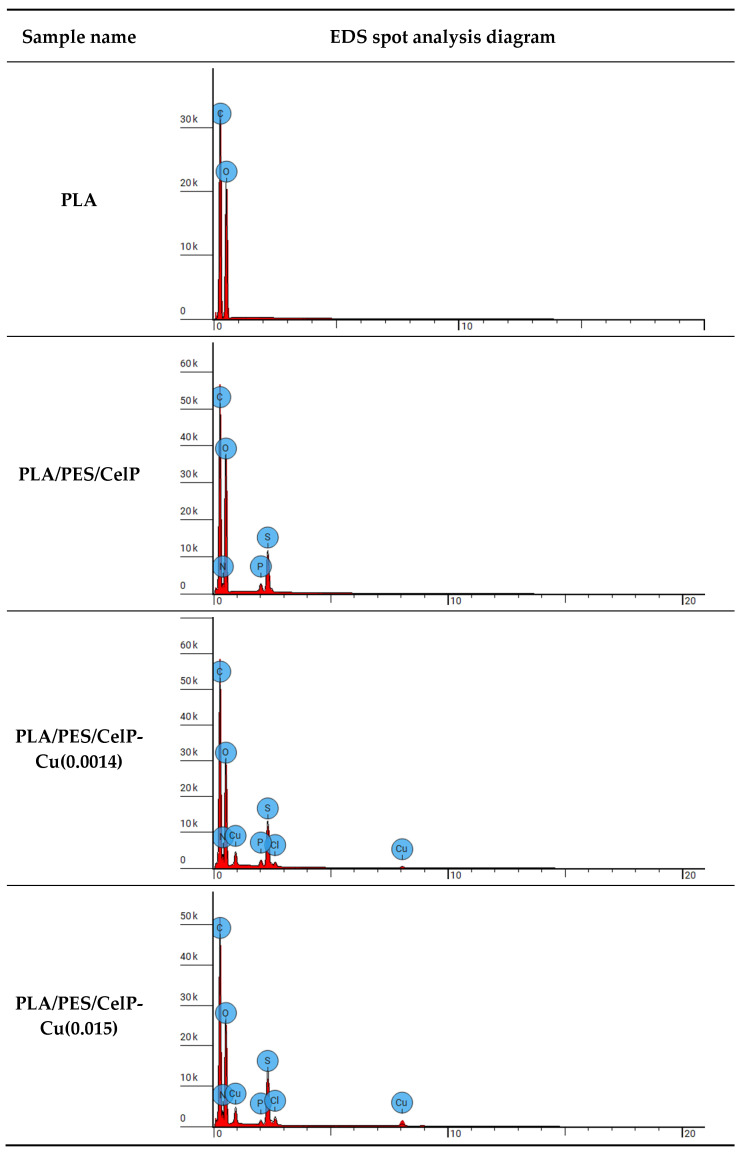
EDS diagrams of PLA and PLA/PES/CelP-Cu composite samples.

Figure 6 shows the EDS spectra of the developed samples. The quantitative results of EDS analysis for the PLA sample and the PLA composite samples are presented in Table 4.

The elemental analysis of the unmodified sample (PLA) displayed the presence of carbon and oxygen, which are the main natural constituents of poly(lactide). After the first step of the dip-coating procedure, additional peaks corresponding to sulfur, phosphorus, and nitrogen were detected. The presence of nitrogen may be explained by the applied solvent, i.e., NMP, which contains nitrogen in its structure. The appearance of the peaks corresponding to sulfur and phosphorus confirmed that the PLA nonwoven was successfully coated with the polymer solution. The sulfur peak originates from the PES present in the coating, while phosphorus peak stems from the cellulose phosphate. After the second step of the modification, two additional peaks connected with the chlorine and copper were observed. This is due to the use of copper (II) chloride in the modifying solution.

The EDS results confirmed the presence of copper in the PLA/PES/CelP-Cu composite samples. The copper content in the samples increases with the concentration of copper in the modifying solution during the dip-coating process, which is consistent with the results obtained from atomic absorption spectrometry.

### 3.2. Biological Characteristics

#### 3.2.1. Antimicrobial Effect

**Figure 7:**
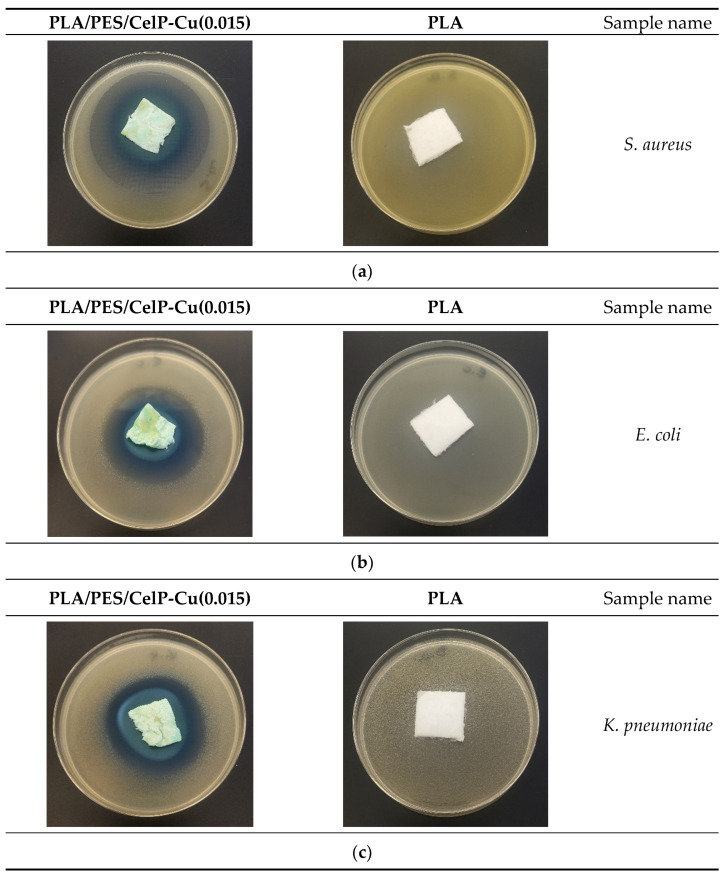

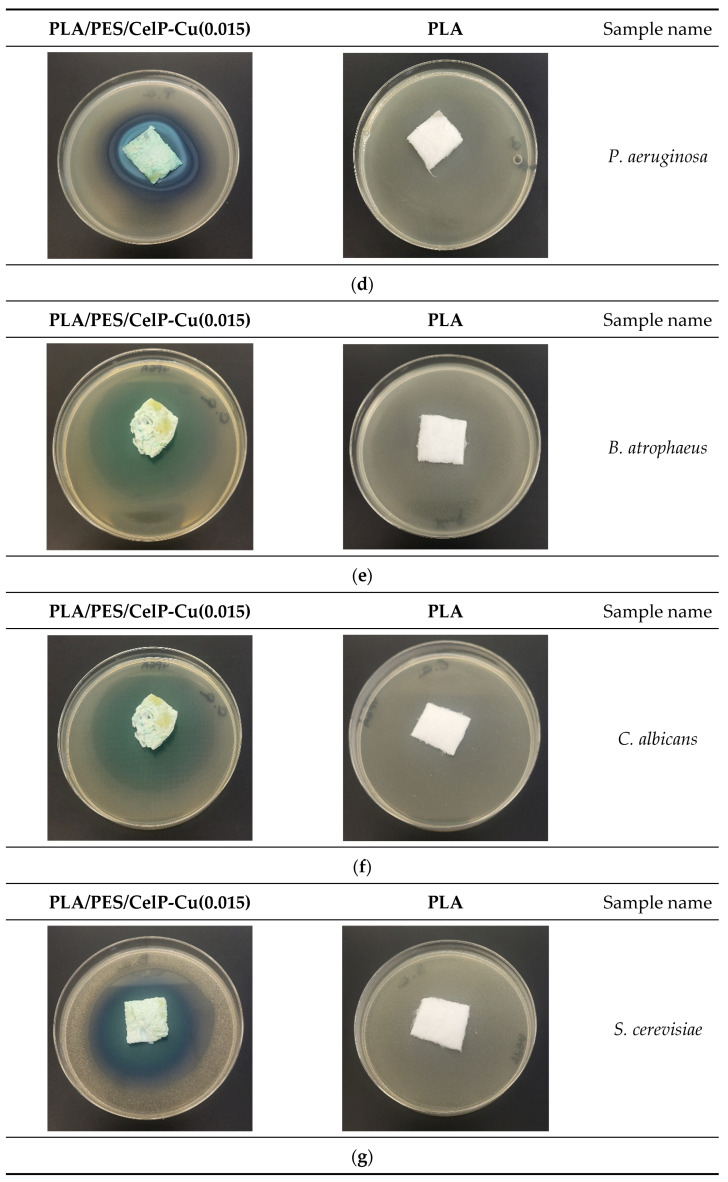

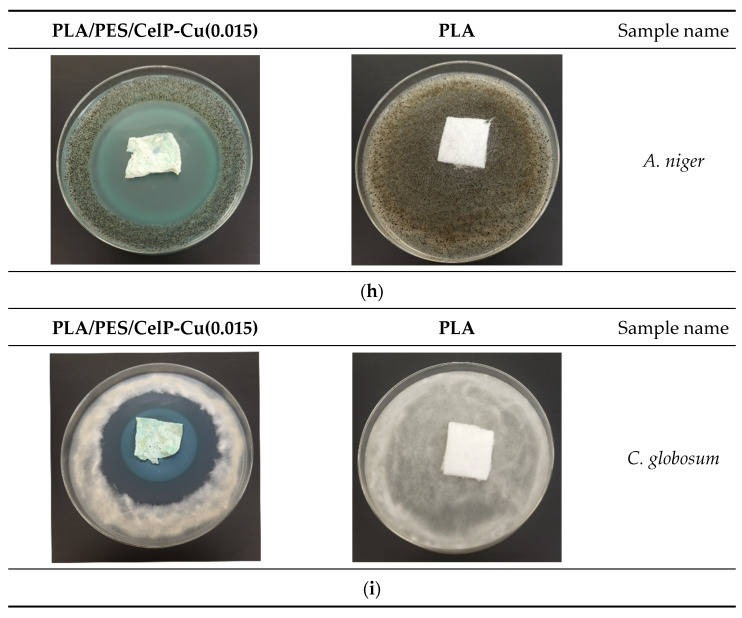
Tests of antimicrobial activity of unmodified poly(lactic acid) nonwoven fabric and PLA/PES/CelP-Cu composites against *S. aureus* (**a**), *E. coli* (**b**), *K. pneumoniae* (**c**), *P. aeruginosa* (**d**), *B. atrophaeus* (**e**), *C. albicans* (**f**), *S. cerevisiae* (**g**), *A. niger* (**h**), *C. globosum* (**i**) inhibition zones of bacterial/fungal growth in *Petri* dishes.

For the unmodified poly(lactide) (PLA) material and the PLA/PES/CelP composite material, which served as control samples, a substantial rise in the number of bacterial and fungal colonies was noticed across the whole surface of the samples. For materials showing no antimicrobial activity (PLA, PLA/PES/CelP), an absent inhibition zone was observed, indicating their inefficiency in preventing microbial growth (Figure 7, Table 5). On the other hand, the PLA/PES/CelP-Cu materials exhibited clear antimicrobial properties, as they effectively inhibited the growth of *S. aureus*, *E. coli*, *K. pneumoniae, P. aeruginosa*, *B. atrophaeus*, *C. albicans*, *S. cerevisiae*, *A. niger*, *C. globosum*, with inhibition zones ranging from 4 to 12 mm (Table 5). While these zones were relatively modest in size, the PLA/PES/CelP-Cu materials demonstrated significant antimicrobial effects in comparison with unmodified fabric. Particularly, the PLA/PES/CelP-Cu(0.015) samples produced the largest inhibition zones, highlighting their superior antibacterial and antifungal activity. These findings, corroborated by the EN ISO 20645:2006 [69] and EN 14119:2005 [70] standards, indicate that the incorporation of copper into the material boosts its ability to suppress microbial growth [45,46]. Figure 6 presents visual evidence of the impact of the PLA and PLA/PES/CelP-Cu samples on the tested bacterial and fungal strains. Each image illustrates the different effects of both the unmodified (PLA) and Cu-coated samples concerning their interaction with the microbes. Analyzing these images provides a comprehensive assessment of the dip-coating process’s effectiveness and its potential implications. The results indicate that the uncoated PLA sample is significantly covered by microbes, while the Cu-coated sample exhibits no such growth, suggesting that the Cu dip-coating process has an inhibitory effect on microbial proliferation.

Some recent studies have demonstrated the promising antimicrobial properties of copper-incorporated PLA composites [45,46,71]. These studies have found that incorporating copper particles or ions into PLA-based materials can effectively inhibit the growth of a wide range of bacterial and fungal species, including clinically relevant pathogens like *S. aureus*, *E. coli*, and *Candida* species. The antimicrobial mechanism is believed to involve the copper ions’ release, which can interrupt the membranes of the microbial cells, interfere with enzymatic processes, and generate oxidative stress [71,72].

The antibacterial and antifungal properties of PLA-Cu composites suggest potential applications as an antimicrobial material. PLA is a multifunctional polymer widely used in different biomedical applications, such as regenerative medicine, tissue engineering, wound healing, and controlled drug delivery. PLA–inorganic hybrids are becoming more important as a result of the established role of metallic nanoparticles in medicine and due to their high antibacterial efficiency, simplicity, and also low cost of technological preparation [73,74,75]. Typical applications of antibacterial PLA composites include wound dressings, medical implants, and protective devices like face masks [76,77,78].

#### 3.2.2. Impact on Plasma Coagulation: Assessment Through aPTT and PT Measurement

**Figure 8:**
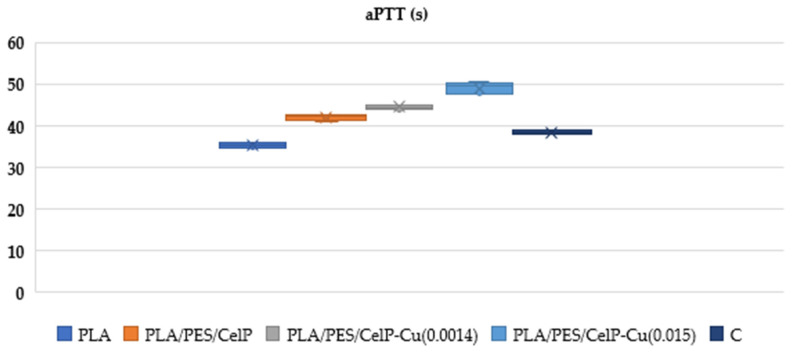
Effect of PLA/PES/CelP-Cu composites on activated partial thromboplastin time (aPTT). The samples: C—plasma control, PLA, PLA/PES/CelP, PLA/PES/CelP-Cu(0.0014), and PLA/PES/CelP-Cu(0.015). The results are presented as mean (×), median (horizontal line), range (bars), and interquartile range (box).

**Figure 9:**
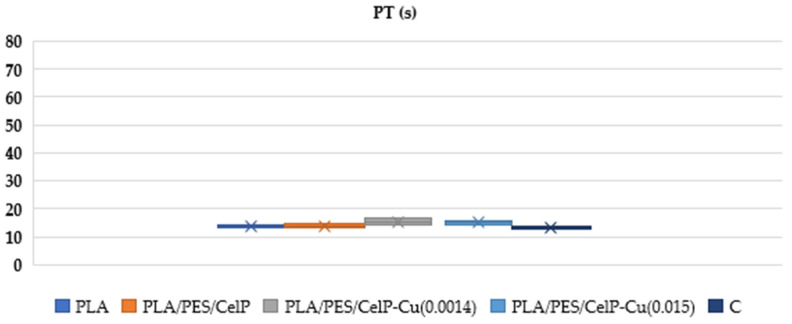
Effect of PLA/PES/CelP-Cu composites on prothrombin time (PTT). The samples: C—plasma control, PLA, PLA/PES/CelP, PLA/PES/CelP-Cu(0.0014), and PLA/PES/CelP-Cu(0.015). The results are presented as mean (×), median (horizontal line), range (bars), and interquartile range (box).

The aim of the research was to assess the potential influence of poly(lactide) nonwoven fabric modifications on the biochemical properties of composite materials, particularly focusing on their influence on blood clotting mechanisms. The modified materials, primarily incorporating copper-coated fibers, were evaluated with respect to two critical coagulation parameters: activated partial thromboplastin time (aPTT) and prothrombin time (PT). These parameters are key indicators applied to evaluate the efficiency of blood plasma coagulation, specifically reflecting the intrinsic and extrinsic pathways of coagulation, respectively [79,80]. Our analysis of the data presented in Figure 8 and Figure 9, as well as the summary provided in Table 6 and Table 7, reveal that the incorporation of copper had a significant impact on these coagulation pathways, indicating that the material modifications influenced the biochemical processes underlying blood clotting. The aPTT in the PLA/PES/CelP-Cu samples reflects the suppression of the intrinsic (contact) coagulation pathway in human blood plasma. These results suggest that Cu may alter the activation of contact factors (XI, XII, HK), leading to a reduction in the levels of these factors in plasma and a resulting prolongation of the aPTT [81]. Particularly, such effects were not observed in PT, suggesting that the effect of copper is restricted to contact factors and does not affect the external components of the coagulation pathway. Although the modified materials exhibited effects on aPTT, their potential advantages, including antimicrobial activity, which were also evaluated in our work, suggest that they could still be suitable for use in clinical settings [82,83].

It should be emphasized that the material modifications do not substantially affect coagulation, indicating their potential suitability for use as wound dressings. Therefore, despite the observed effects of copper on aPTT, the modified materials could be used in clinical settings, considering their additional advantages, such as antimicrobial properties, which were also evaluated in our study [84,85].

## 4. Conclusions

This study successfully developed and characterized poly(lactide) (PLA) composites modified with polyethersulfone (PES) and copper-complexed cellulose phosphate (CelP-Cu) using a pneumothermic melt-blown method followed by dip-coating. The physicochemical, biochemical, and microbiological evaluations demonstrated that the incorporation of CelP-Cu significantly improved the antimicrobial activity of the material while maintaining its coagulation characteristics.

The antimicrobial activity assessment confirmed that the PLA/PES/CelP-Cu composites effectively inhibited the growth of multiple pathogenic microorganisms, such as fungal species, as well as the Gram-positive and Gram-negative bacteria. The presence of copper played a crucial role in microbial suppression. These findings are in agreement with previous studies highlighting the antimicrobial potential of copper-incorporated PLA composites, further supporting their potential use in biomedical applications.

Moreover, the biochemical analysis revealed that the incorporation of copper influenced blood coagulation pathways, as indicated by the prolonged activated partial thromboplastin time (aPTT) in plasma samples. However, no significant effects were observed on prothrombin time (PT), suggesting that the modifications predominantly affected the intrinsic coagulation pathway without impairing the overall hemostatic balance. This is a crucial finding, as it suggests that the material modifications do not pose a high risk of coagulation disorders, which is essential for biomedical applications, including wound dressings and implantable medical devices.

## Figures and Tables

**Table 1:** Processing parameters for the fabrication of PLA nonwovens using the melt-blown technique.

Processing Parameters	
Temperature in zone 1 of the extruder	195 °C
Temperature in zone 2 of the extruder	245 °C
Temperature in zone 3 of the extruder	260 °C
Temperature of the head	260 °C
Temperature of the air heater	260 °C
Airflow rate	7–8 m^3^/h
Mass per unit area of the nonwovens	95 g/m^2^
Polymer yields	6 g/min

**Table 2:** The components of the solutions applied for the dip-coating procedure.

Sample Name	Mixture Components of Film-Forming Solution (%)			
	Polyethersulfone	Cellulose Phosphate	Copper(II) Chloride (1%)	Copper(II) Chloride (10%)
PLA	−	−	−	−
PLA/PES/CelP	+	+	−	−
PLA/PES/CelP-Cu-1	+	+	+	−
PLA/PES/CelP-Cu-2	+	+	−	+

“+” means that the component was in the film-forming mixture and “−” means that it was not.

**Table 3:** Results of the determination of copper content in the PLA/PES/CelP-Cu composite samples by FAAS.

No.	Sample Analyzed	Copper Deposition		Sample Code
		[mg/kg]	[mMol/kg] ^a,b^	
1	PLA	0	0	PLA
2	PLA/PES/CelP	0	0	PLA/PES/CelP
3	PLA/PES/CelP-Cu-1	0.9155	0.0014	PLA/PES/CelP-Cu(0.0014) ^c^
4	PLA/PES/CelP-Cu-2	9.5585	0.015	PLA/PES/CelP-Cu(0.015) ^c^

^a^ Applied molar mass of copper 63.546 g/mol. ^b^ Millimolar concentration. ^c^ Millimolar copper concentration in composite.

**Table 4:** Chemical composition of the PLA and PLA/PES/CelP-Cu composite samples obtained via EDS analysis.

Sample Name	Element Symbol	Element Name	Atomic Conc. [%]	Weight Conc. [%]
PLA	C	Carbon	56.041	48.900
	O	Oxygen	43.959	51.100
PLA/PES/CelP	C	Carbon	53.307	44.645
	N	Nitrogen	4.201	4.104
	O	Oxygen	39.027	43.544
	P	Phosphorus	0.556	1.201
	S	Sulfur	2.909	6.507
PLA/PES/CelP-Cu(0.0014)	C	Carbon	57.160	47.047
	N	Nitrogen	5.943	5.706
	O	Oxygen	32.225	35.335
	P	Phosphorus	0.424	0.901
	S	Sulfur	3.143	6.907
	Cl	Chlorine	0.371	0.901
	Cu	Copper	0.736	3.203
PLA/PES/CelP-Cu(0.015)	C	Carbon	57.570	47.100
	N	Nitrogen	6.496	6.200
	O	Oxygen	30.736	33.500
	P	Phosphorus	0.284	0.600
	S	Sulfur	3.525	7.700
	Cl	Chlorine	0.580	1.400
	Cu	Copper	0.809	3.500

Atomic Conc. [%]—% as a function of the number of atoms; Weight Conc. [%]—% as a function of weight of atoms.

**Table 5:** Results of the antimicrobial activity tests of PLA/PES/CelP-Cu composites.

No.	Sample Name	Average Inhibition Zone [mm]								
		Bacteria and Fungi ^a^								
		Bacteria					Fungi			
		G^+^		G^−^						
		*Ba*	*Sa*	*Ec*	*Kp*	*Pa*	*Ca*	*Sc*	*An*	*Cg*
1	PLA	0	0	0	0	0	0	0	0	0
2	PLA/PES/CelP	0	0	0	0	0	0	0	0	0
3	PLA/PES/CelP-Cu(0.0014)	5	4	6	7	6	6	4	4	6
4	PLA/PES/CelP-Cu(0.015)	7	6	6	7	7	9	8	9	8

^a^ Bacteria and fungi: *An*—*Aspergillus niger*; *Ba*—*Bacillus atrophaeus*; *Ca*—*Candida albicans*; *Cg*—*Chaetomium globosum*; *Ec*—*Escherichia coli*; *Kp*—*Klebsiella pneumoniae*; *Pa*—*Pseudomonas aeruginosa*; *Sa*—*Staphylococcus aureus*; *Sc*—*S. cerevisiae*.

**Table 6:** The impact of the composite materials on aPTT was evaluated for various samples: PLA; PLA/PES/CelP; PLA/PES/CelP-Cu(0.0014); PLA/PES/CelP-Cu(0.015); and C, the control sample.

aPTT					
Sample	PLA	PLA/PES/CelP	PLA/PES/CelP-Cu(0.0014)	PLA/PES/CelP-Cu(0.015)	C
Mean	35.4	42.1	44.57	49.18	38.40
SD	0.63	0.73	0.52	1.37	0.71
Median	35.3	42.25	44.50	49.6	38.40

**Table 7:** The impact of the coated materials on PT was evaluated for various samples: PLA; PLA/PES/CelP; PLA/PES/CelP-Cu(0.0014); PLA/PES/CelP-Cu(0.015); and C, the control sample.

PT					
Sample	PLA	PLA/PES/CelP	PLA/PES/CelP-Cu(0.0014)	PLA/PES/CelP-Cu(0.015)	Control Sample
Mean	13.65	13.90	15.40	15.15	13.1
SD	0.80	1.3	1.60	1.1	0.57
Median	13.7	13.9	15.40	15.2	13.1

## Data Availability

The original contributions presented in this study are included in the article. Further inquiries can be directed to the corresponding author.
